# Personal

**Published:** 1897-09

**Authors:** 


### Personal.

Dr. John F. Raub, medical referee of the U. S. Pension Bureau at Washington, attended the Grand Army of the Republic ceremonies at Buffalo last month, and expressed himself as greatly pleased with the treatment the veterans received at the hands of the residents of the bison city. Dr. Raub is one of the war medalists, having received a bronze medal granted by act of Congress for special valor on the field of battle. He busied himself during a portion of his visit with the investigation of contested or appealed pension cases. After completing his vacation at his old home, Bethlehem, Pa., he will resume his duties at Washington in about a fortnight.

---

Dr. Joseph M. Mathews, president of the Kentucky state board of health and first vice-president of the American Medical Association, attended the meeting of the American Association of Obstetricians and Gynecologists at Niagara Falls, August 17–20, 1897. Dr. Mathews made a speech at the annual dinner of the Association, adding fresh laurels to his already well-rounded reputation as a master of ornate and graceful rhetoric. He also took part in the discussions and contributed to the success of the meeting.

---

Dr. Edward Clark, of 2268 Main street, Buffalo, but whose office is in Ellicott Square, was appointed one of the new milk inspectors, to take effect August 1, 1897. Dr. John H. Grant, of 196 Triangle street, was also appointed milk inspector, to take effect on the same date. These appointments were made from the civil-service list.

---

Dr. Charles E. Sajous, editor of the *Annual of the Universal Medical Sciences*, who has lived for several years in Paris, has returned to Philadelphia, where he will fill the chair of laryngology and act as dean of the faculty of the Medico-Chirurgical College.

---

Dr. Bransford Lewis, of St. Louis, announces the removal of his office to Suite 627 Century Building, Ninth and Olive streets. Hours: 10 A. M. to 1 P. M.; 2 to 3 P. M. Sundays, 9 to 10 A. M.

---

Dr. W. E. Dignen, of Buffalo, who has spent the last few months in Europe in study and travel, has returned and resumed the practice of his profession at 381 Fourteenth street.

Dr. Marcel Hartwig, of 34 East Huron street, Buffalo, is now traveling on the continent of Europe, but will return and resume his professional work in the early autumn.

